# Occupational Therapy for People with Early Parkinson's Disease: A Retrospective Program Evaluation

**DOI:** 10.1155/2022/1931468

**Published:** 2022-07-13

**Authors:** Alan Sadural, Jillian MacDonald, Joelle Johnson, Kavita Gohil, Miriam Rafferty

**Affiliations:** ^1^Shirley Ryan AbilityLab, Chicago, IL, USA; ^2^Rocky Mountain Regional VA Medical Center, Aurora, CO 80045, USA; ^3^Department of Physical Medicine and Rehabilitation, Northwestern University Feinberg School of Medicine, Chicago, IL, USA; ^4^Department of Psychiatry and Behavioral Science, Northwestern University Feinberg School of Medicine, Chicago, IL, USA

## Abstract

**Purpose:**

Clinical practice guidelines establish that occupational therapy (OT) services are indicated for people with early Parkinson's disease (PD). However, OT is uncommon compared to other rehabilitation services. This study describes the development and evaluation of a proactive, consultative OT program for people with early PD as a part of an integrated care approach.

**Materials and Methods:**

The program was developed by an occupational therapist adapting practice guidelines for people with early PD. Retrospective program evaluation occurred at an outpatient rehabilitation clinic. The consultative OT program for early PD includes a 90-minute evaluation with instruction in self-management techniques, individually tailored exercises, and follow-up recommendations. The program was evaluated with the RE-AIM framework. Postprogram semistructured interviews provided patient-reported program effectiveness and satisfaction.

**Results:**

In 2018, 23 individuals used OT out of 77 people with early PD who attended the proactive rehabilitation program. Most individuals (*n* = 16, 69.6%) were within Hoehn and Yahr stages 1-2 and were seen within 3 years of PD diagnosis. Participants presented with deficits in hand strength (60.0 ± 23.4 pounds) and dexterity (right hand 30.0 ± 8.0 seconds) and reported complaints about basic and/or instrumental activities of daily living (*n* = 15, 65.2%). Semistructured interviews (*n* = 16) revealed that most individuals (75%) reported high satisfaction. Of the 10 who recalled a home exercise program, 60% reported continued adherence. Consultative OT was delivered with fidelity in 22/23 individuals (96%). After one year, only two occupational therapists at one clinic had adopted the program, and the program is maintained in the organization.

**Conclusion:**

Occupational therapists reached people in the early stages of PD when a specific program was tailored to evaluate and target their specific needs. Motor activity deficits noted in individuals with early PD support future scaling and sustainability efforts of OT within this population. Quality improvement suggestions are discussed for future implementation and clinical trials.

## 1. Introduction

Occupational performance, or engagement in life activities, is impacted by Parkinson's disease (PD) symptoms, including tremor, bradykinesia, weakness, poor dexterity, fatigue, gait impairment, apathy, depression, and cognitive deficit [1, 2]. An integrated team approach is needed to address these deficits, including contributions from occupational therapists. Clinical practice guidelines (CPG) recommend occupational therapy (OT) services for people with PD in all stages of the disease [3–5]. Yet, data reveals that people with PD rarely use OT [6–8].

Occupational therapy traditionally addresses occupational performance and participation in activities of daily living (ADLs), including self-care, leisure, and work [9]. Occupational therapists are equipped to identify the complex interactions between personal factors, environmental factors, occupational factors, and performance of daily tasks that impact people with PD [10]. Occupational therapy treatment approaches include improving occupational performance by restoring impaired skills or body functions, as well as providing compensatory strategies, including the use of assistive devices, to help the person with PD to adapt to the environment or activity/task [11]. Self-management is enhanced through education on task performance and routine development, as well as patient and care partner training [12]. Furthermore, OT may help improve engagement in life roles within the home, community, and work environments [1].

Several CPG state that OT should be considered early after diagnosis with PD, to provide assessment, education on PD symptoms, and early intervention if ADL difficulties are present [3–5]. Initiating access to OT early after diagnosis can empower people with early PD (PwEP) to improve their disease self-management with more knowledge about the disease course and symptom management [13, 14]. Improvements in self-perceived performance were reported in one example of an individually tailored OT intervention for people across the stages of PD (62% of participants with Hoehn and Yahr stages 1-2) [15]. Early interventions in OT have been proposed to also address quality of life and health management for secondary prevention [16]. Although there is research supporting OT interventions [15], research regarding specific interventions in PwEP and research addressing barriers to health services for these individuals remain scarce [17].

Our purpose is to describe the development and evaluation of a proactive, consultative OT program for individuals in the early stages of PD. We will describe the results of this retrospective program evaluation using the RE-AIM framework, a tool to aid measurement and understanding of evidence-based, implemented programs [18]. RE-AIM is an abbreviation for (1) reach of the program to PwEP and participant characteristics; (2) effectiveness through feedback from PwEP; (3) adoption at the organization; (4) implementation fidelity of the delivery of care; and (5) program maintenance. We conclude with suggestions for future research and quality improvement in OT services for PwEP.

## 2. Materials and Methods

### 2.1. Setting

In 2016, a proactive, consultative rehabilitation program was implemented for PwEP by physical therapists at the Shirley Ryan AbilityLab (SRAlab, formerly the Rehabilitation Institute of Chicago) [19]. Although the program initially focused on PT, PwEP and clinicians expressed interest in OT and speech therapy (ST) services. In late 2017, formal programs in OT and ST were added. Program evaluation interviews and retrospective chart reviews were performed in 2019 for all patients seen in 2018. These methods were determined to be “not human research” by the Northwestern University Institutional Review Board due to the focus on retrospective evaluation of a current clinical program operating using clinical best practices. Due to the observational nature of the evaluation data, we used the Strengthening the Reporting of Observational Studies in Epidemiology (STROBE) checklist to guide reporting (Supplemental Material 1) [20].

### 2.2. Participants

Data were extracted from individuals if they were referred to the proactive PD rehabilitation program for any discipline in 2018 from the University's Movement Disorders Center or the SRAlab Interdisciplinary Parkinson's Disease and Movement Disorders Rehabilitation Screening Program. Individuals with a different movement disorder were excluded from analysis (*n* = 2). In total, 77 PwEP were referred to the proactive PD rehabilitation program, 23 of whom were seen in OT. Of the 77 individuals who utilized the program, 66 were contacted to participate in the program evaluation phone interviews (exclusions: seen in the program prior to 2018, *n* = 8, language barrier, *n* = 2, and error, *n* = 1). Of the 23 OT clients, 16 individuals agreed to participate in the interview (1 opted out following a mailed study letter, and 6 could not be reached with three attempts). These individuals provided verbal consent for audio recording of the interview for program evaluation, future quality improvement efforts, and deidentified dissemination activities.

### 2.3. Implemented Intervention

After a provider referral, PwEP underwent OT, PT, and/or ST using the proactive delivery model. This consultative model includes, at a minimum, a 90-minute evaluation and intervention session with each professional to whom they were referred. This model was shown to be successful in PT [19] and includes a low dose of intervention, focusing primarily on education and development of home programs. An episode of care in the proactive PT model has typically been accomplished in less than four visits [19]. The proactive model was then adapted in OT by a lead occupational therapist (JJ) with 10 years of experience and 7 years of working with people with PD. These adaptations included the addition of OT-specific outcome measures, documentation tips, educational handouts, and other resources. An evaluation plan was developed that focused on functional performance and identification of common motor and nonmotor problems related to PD. The goal of the prolonged initial session was to obtain a comprehensive battery of outcome measures for future comparison and provide immediate exercise recommendations to reduce the need for frequent follow-ups in this mostly independent population. When deficits were noted or reported, an individually tailored plan was created with the patient and therapist through patient empowerment and shared-decision making. Supplemental Material 2 provides a list of the typical evaluation and intervention items considered by the occupational therapist. Evidence-based OT treatments for PwEP primarily address self-management, functional independence, and meaningful occupations [11, 16]. The lead occupational therapist informally trained another occupational therapist in this model of care.

### 2.4. Data Sources

Retrospective data were collected from all PwEP who came through the proactive rehabilitation program (PT, OT, and/or ST) in 2018. Data were extracted from the referring providers' note (neurologist or interdisciplinary clinic screening note) and OT documentation in the electronic medical record (EMR) and managed using a tool created in Research Electronic Data Capture software hosted at the University Clinical and Translational Sciences Institute [21, 22]. Program evaluation phone interviews were completed in 2019 for PwEP seen in 2018.

Reach was measured as the proportion of PwEP who accessed the OT program. Demographics, PD-specific characteristics, and OT evaluation measures are described. Demographics include age, gender, employment status, and insurer. PD-specific characteristics include time since diagnosis and PD severity reported using the Hoehn and Yahr (HY) scale [23]. OT evaluation measures included grip strength, pinch strength, and the Nine-Hole Peg Test. Grip and pinch strength have published normal values for older community-dwelling adults [24]. The Nine-Hole Peg Test is a validated measure of dexterity and upper extremity coordination in PD [25–27]. Additionally, patient-reported basic and instrumental ADL status was categorized by the occupational therapist as independent, modified independent (increased time or use of an assistive device), or requiring assistance.

Program effectiveness was measured using a phone survey and semi-structured interview 12–18 months after the initial evaluation (mean 15 months). Effectiveness was operationalized using the self-reported level of ADL function in everyday tasks after completing consultative OT by rating current status as “improved,” “maintained,” or “declined.” Program satisfaction was measured using a 10-point rating scale. PwEP were also asked if they recalled and continued to use their OT home exercise program (HEP) and other recommendations. The interview guide included multiple choice and open-ended questions, which are shared in the supplementary material (Supplemental Material 3) and provide greater detail of the questions and potential responses/ratings from respondents. Interviews were recorded, and responses were categorized by clinically trained study team members who were not involved in the patient's care (AS; JM). To reduce the risk of bias, an independent auditor (research assistant) not involved in the clinical work checked data entry for accuracy.

Adoption was assessed by the number of occupational therapists trained in the proactive OT model, the number of sites within the organization adopting its use, and the number of referrers to the proactive OT program. These data were extracted from administrative data.

Implementation fidelity was measured by describing the extent to which the number and frequency of visits match a proactive, consultative model (e.g., 1–4 visits spread out to facilitate independent exercise completion). Fidelity measures include treatment information, education, and provision of an OT HEP supported by best practice recommendations [11].

Maintenance of the program is described as the number of PwEP seen in the early OT program in 2020 based on administrative data, although the program evaluation focused on the first year of the program (2018).

### 2.5. Statistical Analysis

Demographics, OT assessment, and phone interview data are presented with descriptive statistics. The categorical outcome data were presented as *N* (%) and continuous data as means ± standard deviations. We compared PwEP who received OT to the other recipients of the proactive rehabilitation program (PT and/or ST) using Chi-square tests for categorical data and *t*-tests for continuous data, after checking normality assumptions, at a significance level of 0.05.

## 3. Results

### 3.1. Reach

**Figure 1:**
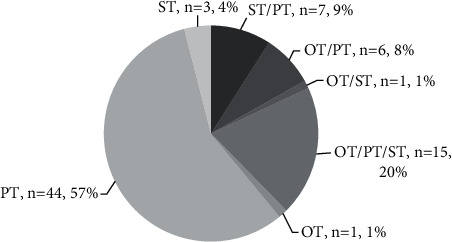
Distribution of all PwEP seen in 2018 (*n* = 77). Number of disciplines seen by each patient. *Note*. OT = occupational therapy; PT = physical therapy; PwEP = people with Parkinson's disease; ST = speech therapy.

Of the 77 people seen in the proactive rehabilitation program during 2018, 23 PwEP (30%) were seen by OT. Out of 23, 22 were seen in combination with at least one other discipline (Figure 1). Demographic and PD-specific characteristics of PwEP who saw an occupational therapist under the consultative delivery model are listed in Table 1. There were no differences found between the group of people who accessed OT compared to those who accessed PT and/or ST, except that there was a significantly smaller proportion of OT users with unknown ethnicity listed in the medical record (*p*=0.0213).  Table 2 describes OT evaluation measures and treatment information. The OT evaluation measures are presented in comparison to average normative data from healthy age-matched men and women and suggest that mild impairments were present in the PwEP participating in OT. In all test variables for both men and women, the values of grip and pinch strength were consistently weaker, and the time to complete the Nine-Hole Peg Test was consistently slower in the study sample compared to health age-based matched sample data. The only exception to this is key pinch strength on the right for women, with the study sample averaging 13.6 (±1.5) pounds pinched versus 13.4 (±2.8) pounds pinched in the healthy, age-based matched sample. Additionally, most individuals (*n* = 20, 87.0%) were documented at a level of modified independence for at least one basic ADL. The top five reported problem areas were upper extremity dressing (*n* = 19, 82.6%), eating (*n* = 18, 78.2%), lower extremity dressing (*n* = 18, 78.2%), bathing (*n* = 16, 69.6%), and grooming (*n* = 16, 69.9%). The two most frequently reported problems with instrumental ADLs were writing (*n* = 9, 39.1%) and keyboarding (*n* = 7, 30.4%).

### 3.2. Effectiveness

Sixteen of the 23 PwEP who engaged in the OT program completed interviews. Over a year after participation in the consultative model of OT, the level of function in everyday tasks was self-reported as “maintained” in 11 respondents (68.8%), “improved” in one (6.2%) respondent, and “declined” in four respondents (25.0%). Twelve (75.0%) respondents were highly satisfied with the OT program. Ten (62.5%) respondents were able to recall their HEP, but 4/10 stated that they no longer follow the exercises. Nine (56.3%) respondents recalled and described OT recommendations they had utilized, the most common being ADL tips, writing strategies, and computer keyboard modifications.

### 3.3. Adoption

Two occupational therapists conducted proactive evaluation and OT intervention sessions in 2018. All proactive OT sessions were conducted at a single site, even though the proactive PT program spread to two other affiliated outpatient clinics in the same timeframe. Six physicians (neurologists and physiatrists) referred PwEP to the OT program in 2018, out of 15 total referrers to all disciplines providing this consultative model.

### 3.4. Implementation Fidelity

All PwEP in the OT program were within five years of diagnosis. Three OT delivery patterns were used: (1) 17 (74%) PwEP completed one 90-minute session with no additional follow-up, (2) five (22%) PwEP completed 1–3 additional follow-up visit(s), and (3) one (4%) individual had a restorative bout of OT with seven follow-up visits. Either all PwEP were recommended to attend a re-evaluation in 6–12 months to document functional status and monitor HEP, or the occupational therapist documented that the individual would contact their physician if further treatment was needed.

Table 2 presents results on the assessment tools used and education provided during the initial consultative visit, reflecting implementation fidelity. All PwEP were educated in OT and the role of this provider as an integrated healthcare team member. The outcomes of specific assessment measured are presented in 23 PwEP (100%) who performed grip strength and the 9-Hole Peg Test and 20 PwEP (87%) who performed pinch strength testing. In this table, 19 (83%) PwEP received a proactive home exercise program, including fine motor coordination, upper extremity active range of motion, and strength. Additional common topics of education were home safety modifications and expected PD symptoms in anticipation of disease progression. Less commonly, patients received education on equipment aids to promote occupational performance (30.4% of the time).

### 3.5. Maintenance

The program was sustained for the duration of 2018 and continues to reach PwEP into 2022 with adaptations to the initial program. Adaptations have included new organizational leadership support of OT program champions and mentorship opportunities. In 2020, programmatic reach grew by 47% from 23 to 34 PwEP. Over time, adoption spread to 2 sites, 4 occupational therapists, and 11 referrers.

## 4. Discussion

This program evaluation offered insights into a novel opportunity for occupational therapists to reach individuals with early stage PD. Most individuals with early PD chose to engage in this consultative model of care with just one 90-minute session focused on assessment, education, and exercise prescription. People with early PD who attended this model of OT presented with impairments in dexterity and hand strength compared to the similarly aged healthy population. Additionally, 87% of PwEP reported difficulty with at least one ADL. The observed impairments in hand strength and dexterity may be related to the self-reported difficulty or slowness with ADLs, suggesting the need for greater use of OT interventions early after diagnosis [28]. It is important for these mild deficits to be addressed through exercise, education on compensatory strategies that can improve related functions, and long-term monitoring.

At this time, there is a lack of clarity around which treatments will best target the mild deficits noted in people with early PD. Foster and colleagues hypothesize that individuals in the early stages “may benefit greatly from interventions that promote the integration of self-management habits and other healthy performance patterns…into daily life” [11]. Future work on assessments and OT treatments for PwEP should adopt a framework, such as the Person-Environment-Occupation-Performance (PEOP) Model, or use other lifestyle management theories to better guide a person-centered approach [10, 29]. An OT framework would help to target this population with mild impairments and unique occupational performance concerns, which may include disease self-management, tasks required for job retention (e.g., keyboarding), and traditional ADLs. In this current work, a clinician adapted the evidence to their current organizational workflow and documentation constraints. Applying the PEOP framework could support a more comprehensive evaluation and plan of care. However, the program setup would take additional time and resources, such as addition of new measures to the electronic medical record template. Occupational therapists who work with people with PD are key stakeholders to apply these frameworks and should be supported through compensated time or other incentive programs for the development of novel consultative programs for PwEP.

In this described program, OT provided baseline functional assessments, individually tailored exercise programs and environmental modification recommendations, and basic and instrumental ADL management strategies. The occupational therapist recommended treatment of observed deficits. The majority of PwEP in the OT program (74%) required a low-dose delivery model, with only one 90-minute consultative session. This delivery model had high program satisfaction, suggesting good value without the need for a burdensome commitment. People in the early stages of PD may be more interested in this consultative model versus a more extensive approach for a variety of factors, such as the individual's mild disease severity, insurance, time, or cost. Additional research is needed to better understand the best delivery models and assessments of low-dose, consultative models of care [30].

Improving reach, adoption, and effectiveness of OT for PwEP are opportunities for clinical and research improvement. We found OT to be the least common area of rehabilitation, which aligns with data from larger patient registries [8]. Similar to Roberts and colleagues, we found that OT was initiated most frequently in the context of multidisciplinary rehabilitation rather than a solo entry point into the rehabilitation system [8]. We also found that follow-up visits were rare within the first year, despite the documented impairments supporting the need for OT. Incorporating relevant screening measures in hand strength, dexterity, or daily living self-assessments such as the Movement Disorders Society revision of the Unified Parkinson's Disease Rating Scale [31] in neurology clinics may promote earlier referral and treatment in OT. In addition, further study on successful interventions to improve hand strength and dexterity in PD could improve education provided to the physician and PwEP regarding the need for proactive OT programs, as well as OT delivery itself.

Additional opportunities to improve proactive OT delivery include exploration and application of OT interventions as they relate to employment and self-management. Our data suggest that our initial proactive OT program implementation focused on traditional areas of basic and instrumental ADLs. However, approximately 35% of PwEP in this program were working full or part time, and computer use strategies were a commonly recalled tip from participants. Proactive OT can tailor interventions to address employment, including the psychosocial implications of working with a chronic condition, work-related skills, and workplace productivity. Energy conservation techniques and self-management education may help manage symptoms to help PwEP stay in the workforce longer [32, 33]. Further research to advance PD-specific self-management training programs may improve proactive OT delivery [34]. For instance, it has been proposed that OT can play a role in developing a health plan to promote a healthy lifestyle in older adults using methods that could apply to PwEP [29].

### 4.1. Limitations

The results of this program evaluation have limited generalizability. First, the sampling strategy for this single-site evaluation introduced recall and selection bias due to the small sample size, delayed follow-up, and lack of control. Second, the high level of variability in clinical documentation made it difficult to extract data from the EMR. Additional organizational support for OT-specific program development and facilitation could improve implementation fidelity in the future. Some documentation had evaluation items missing, which could be due to clinician time constraints or patient barriers, such as cognition or language. Third, our cohort was fully insured, primarily white, and English-speaking, which may limit generalizability to more diverse clinics. Lastly, the nature of a single consultative visit did not allow for follow-up assessments to document the clinical effectiveness of the proactive OT model beyond satisfaction. Future research should include regular follow-up assessments in order to compare the results of this intervention to other OT interventions.

## 5. Conclusion

This OT program evaluation revealed that even individuals with early PD have key occupational performance and participation issues that can be addressed by occupational therapists. While the use of OT early after diagnosis was lower than other disciplines, satisfaction remained high for those who participated. Future quality improvement efforts, aided by programmatic support or implementation research funding, are recommended to improve the reach of this program.

## Figures and Tables

**Table 1:** Demographic and Parkinson's disease-specific characteristics (*n* = 23).

	All (*n* = 71)	OT (*n* = 23)	PT and/or ST without OT (*n* = 48)	Statistical significance
Gender (% male)	44 (62%)	15 (65.2%)	29 (60.4%)	0.6966
Age, year	66 ± 9	68 ± 11	65 ± 8	0.1085
Race				0.3755
White	26 (36.6%)	12 (52.2%)	14 (29.8%)	
Black	1 (1.4%)	0 (0%)	1 (2.1%)	
Asian	3 (4.2%)	1 (4.3%)	2 (4.3%)	
Others	12 (16.9%)	2 (8.7%)	10 (21.3%)	
Unknown (declined to answer)	28 (39.4%)	8 (34.8%)	20 (42.6%)	
Ethnicity				0.0213^*∗*^
Hispanic	0 (0%)	0 (0%)	0 (0%)	
Non-Hispanic	44 (62%)	19 (82.6%)	25 (54.4%)	
Unknown (declined to answer)	25 (35.2%)	4 (17.4%)	21 (45.7%)	
Insurance				0.1598
Medicare with secondary insurance	27 (38%)	11 (47.8%)	16 (33.3%)	
Medicare only	3 (4.2%)	2 (8.7%)	1 (2.1%)	
Private insurance only	41 (57.7%)	10 (43.5%)	31 (64.6%)	
Employment status				0.1476
Full time	16 (22.5%)	7 (30.4%)	9 (18.6%)	
Part time	1 (1.4%)	1 (4.3%)	0 (0.0%)	
Retired for age	23 (32.4%)	8 (34.8%)	15 (31.3%)	
Retired for disability	2 (2.8%)	1 (4.3%)	1 (2.1%)	
Unemployed	1 (1.4%)	1 (4.3%)	0 (0.0%)	
Missing	28 (39.4%)	5 (21.7%)	23 (47.9%)	
Hoehn and Yahr stage				0.4565
1	11 (15.5%)	4 (23.5%)	7 (33.3%)	
2	26 (36.6%)	12 (70.6%)	14 (66.7%)	
3	1 (1.4%)	1 (5.9%)	0 (0.0%)	
Missing	39 (54.9%)	6 (26.1%)	33 (68.8%)	
Time since Parkinson's disease medical diagnosis				0.2744
1–12 months	41 (57.7%)	12 (52.2%)	29 (60.4%)	
12+ months–3 years	14 (19.7%)	4 (17.4%)	10 (20.8%)	
3+ years	12 (16.9%)	5 (21.7%)	7 (14.6%)	
Missing	4 (5.6%)	2 (8.7%)	2 (4.2%)	

^*∗*^Significance of <0.05.

**Table 2:** OT evaluation measures and treatment information (*n* = 23).

OT evaluation measures (average (SD))	Study sample (men = 69.7 ± 11.2 yrs; women = 65.9 ± 10.7 yrs)		Comparison to healthy age-matched samples from literature [24, 26, 27]	
	Men (*n* = 15)	Women (*n* = 8)	Men	Women
Hand strength (*n* = 23)				
Grip left (lbs.)	71.1 (19.6)	42.0 (17.3)	83.8 (17.6)	50.7 (11.2)
Grip right (lbs.)	70.7 (19.2)	40.6 (18.9)	88.2 (18.3)	52.9 (11.7)
Finger strength (*n* = 20)				
Key pinch left (lbs.)	18.8 (4.8)	12.5 (1.9)	20.9 (5.0)	12.6 (2.5)
Key pinch right (lbs.)	19.1 (4.5)	13.6 (1.5)	22.3 (4.3)	13.4 (2.8)
Palmar pinch left (lbs.)	16.1 (5.1)	11.2 (3.6)	19.2 (4.4)	12.7 (2.9)
Palmar pinch right (lbs.)	16.5 (5.0)	12.1 (3.5)	19.5 (4.7)	13.5 (3.2)
Nine-Hole Peg Test (*n* = 23)				
Left hand (seconds)	30.3 (6.1)	27.7 (6.8)	22.3 (3.71)	21.4 (5.66)
Right hand (seconds)	29.9 (8.5)	29.8 (7.5)	21.2 (3.29)	19.9 (3.15)
Chief complaints reported to OT (*n* = 23)				
Fine motor coordination and control	14 (60.9%)			
Upper extremity strength and endurance	10 (43.5%)			
Basic and instrumental ADLs	15 (65.2%)			
ADLs	3 (13%)			
Tremors	11 (47.8%)			
Balance and mobility				
OT education information (implementation fidelity) (*n* = 23)				
Proactive OT education topics				
Role and purpose of OT	23 (100%)			
Home exercise program	19 (82.6%)			
Adaptations and modifications	16 (69.6%)			
Condition information	8 (34.8)			
Equipment and device use	7 (30.4%)			
Barriers to education				
Cognitive deficits	2 (8.7%)			
Language	1 (4.3%)			
Memory deficits	1 (4.3%)			

## Data Availability

The data used to support the findings of the study can be obtained from the corresponding author upon reasonable request.

## Supplementary Materials

Supplementary Materials Supplemental Material 1. STROBE statement: completed checklist for Strengthening the Reporting for Observational Studies in Epidemiology. Supplemental Material 2. Occupational therapy (OT) evaluation and intervention: a list of typical evaluation and intervention items from the consultative OT session. Supplemental Material 3. Phone survey and semistructured interview discussion topics.
