# Supplementary material for: Occupational Therapy for People with Early Parkinson's Disease: A Retrospective Program Evaluation
Source: Parkinsons Dis. 2022 Jul 13;2022:1931468. doi: 10.1155/2022/1931468 (PMC9300278; doi:10.1155/2022/1931468)
Supplement: Supplementary Materials — Supplemental Material 1. STROBE statement: completed checklist for Strengthening the Reporting for Observational Studies in Epidemiology. Supplemental Material 2. Occupational therapy (OT) evaluation and intervention: a list of typical evaluation and intervention items from the consultative OT session. Supplemental Material 3. Phone survey and semistructured interview discussion topics. [file 1931468.f1.zip › 1931468.f1/Supplemental Material 2.docx]

**Supplemental Material 2.** **Occupational Therapy (OT) Evaluation and Intervention:** A list of typical evaluation and intervention items from the consultative OT session.

| Evaluation Tools | Intervention |
| --- | --- |
| Medical History, Onset of PD, Symptoms | |
| - Parkinson’s Disease Questionairre PDQ-39 - Review Medical Screening Form - Guage person’s knowledge of OT | - Review Session - Education on OT and its role in PD - Discuss adherence to medical recommendations and follow up |
| Occupational Profile and Performance Deficits | |
| - ADLs: Rate & review eating, grooming, bathing, dressing, toileting, transfers | - Task specific activities - Education in large amplitude movement - Compensatory techniques as needed - Special considerations (sexual activities, medication management) - Health and life management and maintenance - Safety and emergency management - Shopping - Communication and technology - Driving and community mobility - Refer out as needed. - Adaptive equipment considerations |
| - IADLs: Rate & review meal preparation, writing, keyboarding, phone use, grocery shopping, clothing care, cleaning, transportation, medication management |  |
| - Work | - Considerations of reporting at work - Job performance - Task specific activities - Compensatory techniques as needed - Fatigue management - Movement on the job - Cognitive considerations - Ergonomics |
| - Social Participation | - Life management tools to increase participation |
| - Leisure | - Exercise routine modification - Life management tools |
| Physical Performance | |
| - Strength   - Grip strength   - Pinch Strength   - Manual Muscle Test - Range of motion | - Large amplitude movement - Home exercise program - Cardiovascular exercise - Handwriting - Tremor management |
| - Fine Motor Coordination   - Subjective report on difficulties with IADs (see previous section)   - 9 hole peg test |  |
| - Vision assessment   - Pursuits   - Convergence | - Education - Home exercise program |
| Non-Motor Issues | |
| - Fatigue & sleep   - Parkinson’s Disease Fatigue Scale | - Education and resources - Encourage exercise - Education on sleep hygiene - Track sleep as needed |
| - Mental and Emotional Well-being | - Breathing strategies - Education and resources - Referral to appropriate provider |
| - Functional Cognition | - Education - Collaboration with Speech Therapist |
| - Pain | - Address as needed |
