# Supplementary material for: Occupational Therapy for People with Early Parkinson's Disease: A Retrospective Program Evaluation
Source: Parkinsons Dis. 2022 Jul 13;2022:1931468. doi: 10.1155/2022/1931468 (PMC9300278; doi:10.1155/2022/1931468)
Supplement: Supplementary Materials — Supplemental Material 1. STROBE statement: completed checklist for Strengthening the Reporting for Observational Studies in Epidemiology. Supplemental Material 2. Occupational therapy (OT) evaluation and intervention: a list of typical evaluation and intervention items from the consultative OT session. Supplemental Material 3. Phone survey and semistructured interview discussion topics. [file 1931468.f1.zip › 1931468.f1/Supplemental Material 3.docx]

**Phone Survey and Semi-Structured Interview Discussion Topics**

**First participants were asked about their overall experience. Then, those who also participated in occupational therapy were asked occupational therapy specific questions. Some questions were multiple choice or ordinal scales, while others were open-ended with probes.**

| **General Questions** | | |
| --- | --- | --- |
| **Topic** | **Question** | **Follow up Discussion** |
| **Satisfaction** | On a scale from 0 to 10, with 0 being not beneficial at all, and 10 being extremely beneficial, how would you rate your overall experience? | What made the experience (so/more/less) beneficial?  *If they saw multidisciplines:* Would you say you found one discipline to be more or less beneficial and if so, why?  *If they saw mutlidisciplines*: How did you time your visits with the different therapists: Did you see them all around one time, or did you see them spread out? If so, why? *Was it your choice or staff availability?* |
|  | On a scale from 0 to 10, with 0 being not at all likely, and 10 being extremely likely, how likely are you to recommend the program to another person you meet with early Parkinson’s disease? | *If they saw multidisciplines:* Would you give different recommendations for the different disciplines, and if so, what would they be? |
| **Process** | In the time leading up to your first therapy session, did you have any problems with scheduling, insurance, or your arrival to SRALab? | If so, what could have been done to make the experience easier for you? |
| **Care Path** | Did you choose to follow up with the therapist for more than a single evaluation visit? | And if so, did you have the right amount of follow ups for what you felt you needed?  *(If yes):* What was beneficial about the follow ups?  *(If no):* Would you have preferred more or less follow ups?  Would you have liked more phone or physical follow ups?  Did you ever feel overwhelmed by the amount of information you were given, and if so do you have suggestions for improvement?  *(If yes … and if multidiscipline were seen, then)* Did seeing multiple therapists contribute to feeling overwhelmed and if so, what do you think your therapy team could have better? |
| **Resources** | Your therapist(s) provided many resources such as handouts or exercise instructions. Which of them were the most helpful and do you have any suggestions for improvements? | Which of them were the most helpful and do you have any suggestions for improvements? |
| **Cost** | Do you recall having any problems related to the cost of the program? | *(If yes):* Did you have difficulty with insurance coverage?  *(If yes):* Did you find cost to be a barrier in attending the program or in following through with the home exercise program? |
| **Occupational Therapy Specific Questions** | | |
| **Occupational Therapy** | What exercises are you doing at home that your OT gave you and how much time do you spend doing them each week?  Since completing OT have your function in everyday tasks improved, been maintained or declined?  Did your therapist recommend any equipment to use with everyday tasks/exercise? | If so, did you purchase any of them and are you using them?  Are you using any “tips and tricks” from your occupational therapist? *(handwriting strategies, dressing strategies, etc.)*  What were the best parts of occupational therapy?  What, if any, parts of occupational therapy would you recommend we do differently? |
| **Process** | Did one of your therapists ever refer you to other healthcare providers, such as a social worker, psychologist, vocational rehabilitation specialist? | If so, which ones? |
| **Care Path** | In regards to long -term follow up, have you see either your PT, OT or Speech therapist back after you were originally discharged? | If yes, have you found this to be helpful?  If no, what have been the barriers to accessing long term follow up with this member of the team? |
